# Fairness in scientific publishing

**DOI:** 10.12688/f1000research.10318.2

**Published:** 2017-01-17

**Authors:** Philippa C. Matthews

**Affiliations:** 1 Nuffield Department of Medicine, University of Oxford, Oxford, UK; 2 Harris Manchester College, Oxford, UK; 3 Department of Infectious Diseases and Microbiology, Oxford University Hospitals NHS Foundation Trust, John Radcliffe Hospital, Oxford, UK

**Keywords:** Academic publishing, peer review, impact factor, metrics, data visualization, open access

## Abstract

Major changes are afoot in the world of academic publishing, exemplified by innovations in publishing platforms, new approaches to metrics, improvements in our approach to peer review, and a focus on developing and encouraging open access to scientific literature and data.

The FAIR acronym recommends that authors and publishers should aim to make their output **F**indable, **A**ccessible, **I**nteroperable and **R**eusable. In this opinion article, I explore the parallel view that we should take a collective stance on making the dissemination of scientific data *fair* in the conventional sense, by being mindful of equity and justice for patients, clinicians, academics, publishers, funders and academic institutions.

The views I represent are founded on oral and written dialogue with clinicians, academics and the publishing industry. Further progress is needed to improve collaboration and dialogue between these groups, to reduce misinterpretation of metrics, to minimise inequity that arises as a consequence of geographic setting, to improve economic sustainability, and to broaden the spectrum, scope, and diversity of scientific publication.

## Introduction

Substantial and positive changes are currently underway in academic publishing; we now have the important opportunity to explore the many potential benefits that can stem from new ways to disseminate scientific data ^1^. Despite the improvements that are emerging, managing a piece of work from conception to publication can be a long and complicated journey, and elements of the process may often feel ‘unfair’.

Advocates of data dissemination encourage aspiration to the principles enshrined in the ‘FAIR’ acronym; work should be **F**indable, **A**ccessible, **I**nteroperable and **R**eusable ^2^. As well as endorsing these attributes, I here represent the view that we must also develop a collective responsibility to make data sharing *fair* in the conventional sense; the way we generate, represent, review, share and use data should be underpinned by justice.

Recent discussions around the dissemination of my own data led me to seek opinion from a cross-section of colleagues within academic medicine. To formalize this exercise, I used an online questionnaire and then followed this up with a parallel approach to seek feedback from the publishing industry. This piece is a representation of some of the key themes that arose as a result of the two-pronged questionnaire, presentations at publishing and data visualization meetings, and ongoing dialogue throughout. The opinion that I present here is my own, but is underpinned by this varied input, with the aims of prompting further discussion, building bridges between publishing and academia, and advancing constructive dialogue to inform future progress.

## Questionnaire methods and results

Questionnaires were posted on-line at https://www.surveymonkey.co.uk/. The methods and entire datasets collected from the quantitative and qualitative input submitted by 102 academics and 37 representatives of the publishing industry, are available to view and download as PPT files from 3 and 4 respectively; the questionnaire metadata can also be accessed in full from the Oxford University Research Archive ( https://doi.org/10.5287/bodleian:J5aekGAMy).

This does not aspire to be a formal scientific study: the feedback I have collated represents individual opinion and the resulting work is my own personal synthesis of this dialogue and experience.

## Domains for discussion in academic publication

### Timelines

Conventional publication timelines commonly amount to weeks or months consumed by submission, peer-review, editorial decisions, potential corrections and resubmission ^5^. Over 70% of academic survey respondents, agreed with the statement ‘I am frustrated by the length of time it takes to publish my work’ ^3^, and over 80% of publishers agreed that reducing the timelines involved in academic publication should be a ‘crucial priority’ ^4^. Such delays can stifle scientific progress in a variety of ways. Over the long time courses of publication, data frequently decay such that they are out of date before they go to press ^6, 7^. Delay also leads to academic paralysis: until their work is published, academics may refrain from presenting or discussing their data publicly, thereby limiting its impact, impeding developments and collaborations, and allowing flaws and discrepancies to go unchallenged. There is also personal paralysis, whereby delays limit the next phase of an existing body of work, reduce the likelihood of moving on to a new project, and impinge on recruiting a team, applying for academic jobs, or securing funding ^3, 7^.

Reducing delays is an important aspiration but one that comes with practical caveats. One publisher says: ‘Timeliness is important. So is quality control. The latter negatively impacts the former’ ^4^. In conventional models of publishing this may have been the case, but we should now strive to dismantle the view that long delays are an inevitable consequence of producing high quality output. Happily, this framework is shifting as a result of parallel improvements in allowing academics to post their own work online, and in new approaches to post-publication peer review, an approach that has been adopted as an inventive compromise to reduces delays and promote data sharing, without sacrificing a quality assurance framework (e.g. by the F1000 and Wellcome Open Research platforms at https://f1000research.com, https://wellcomeopenresearch.org/) ^1, 8^. Reorganisation of the publication process has already contributed to reducing delays in other ways: authors now have the option of disseminating their work through pre-publication archives (e.g. BioRxiv, http://biorxiv.org/) or on data-sharing platforms (e.g. Figshare, https://figshare.com/).

### Peer review

Peer review is intended to provide quality assurance, a principle that is of universal importance to all stakeholders. Asked to respond to the statement ‘peer review functions equitably and contributes to improving the quality of my work’, 58% of academics agreed ^3^. However, there is universal recognition of the potential pitfalls of such a process: a reviewer may not be impartial, may be less expert than the authors of the work for which they are providing critique, may not give the task the time that it deserves, and may – on occasion – just get it wrong ^9^. There can also be concern, as stated by one academic, that ‘creativity is stifled in this process’. On these grounds, peer review has continued to be accepted only as the ‘least worst’ model ^9^.

However, many improvements to peer review are evolving, with support and enthusiasm from both academics and publishers ^3, 4^. These include:

- Making peer reviews open access (e.g. F1000, https://f1000research.com and PeerJ, https://peerj.com/), or providing double-blind peer review ^9^;
- Using structured formats or templates for critical review, and developing collaborative peer review so that a consensus opinion is provided by a team (e.g. Frontiers, http://home.frontiersin.org/);
- Promoting a model that seeks online feedback from the entire scientific community (now a component of many open access review systems, including those at https://f1000research.com);
- Asking reviewers to suggest additional experiments only when these are deemed essential to the work and can be conducted within an agreed time frame (e.g. eLife, https://elifesciences.org/);
- Ensuring that publishers and journals consistently apply a set of criteria to ensuring reviewers have appropriate expertise to critique an article (e.g. F1000, https://f1000research.com);
- Improving editorial oversight and influence to ensure the process is conducted fairly and to arbitrate in cases where there is conflict of opinion.

Recognition for the substantial contribution made by reviewers is important, and strides forward are afoot in providing formal acknowledgement of the body of work undertaken by reviewers. Reviews themselves are becoming independently accredited pieces of scientific work that are a recognised part of a formal academic portfolio (including visibility on ORCID, http://orcid.org/), can be ranked and rated, are published with a DOI to make them accessible and citable, and can lead to the award of CME points ^10, 11^. Reviewers can now log this activity in a systematic way (e.g. using Publons, https://home.publons.com/).

### Barriers to communication

Much of the communication between academia and publishers is uni-directional and undertaken via rigid online portals, potentially leading to frustrations on both sides. Less than a quarter of academic respondents agreed or strongly agreed that they would feel ‘comfortable and confident contacting editors and publishers to discuss work before submitting for publication’ and only one in three reported having experienced positive interactions of this kind ^3^. Interestingly, academics’ views on this point also reflect a degree of uncertainty about whether discussion with editors and publishers is appropriate at all: they raise concerns that this amounts to ‘coercion’ or is in some way ‘cheating’ the system ^3^.

Collective responses to how communication should be improved include the need for improving formal and public interdisciplinary discussion, as well as the more personal view from academics, who are keen for editors and publishers to provide a reliable and named point of contact. There is also a collective responsibility for both parties to commit to effective communication, recognizing the ways in which appropriate dialogue can improve the content or accessibility of scientific output, and encouraging routine and transparent dialogue between publisher and academic.

### Metrics

The impact factor, the most widely quoted metric, has disproportionate influence over the behaviour of academics, despite never being designed as a measure of the quality of any given piece of work ^5^. To quote one publisher, impact factor is ‘embedded in researcher culture’ ^4^. However, there has been increasing recognition that the metrics of any individual piece of work should be of more importance than those of the journal in which it is published, and that we should move away from assessing ourselves, or each other, based on this criterion ^7, 12^. It is also important to be mindful that citations can be relatively easy to amass for articles written on major topics, while equally rigorous work in a more niche discipline naturally attracts a smaller audience.

‘The impact factor is broken’ stated one academic medic ^3^. Only 19% of publishers disagreed with this statement, and others added their own descriptions of the impact factor as ‘misused and outdated’, ‘obsolete’ and ‘a horrible obsession for editors and authors’ ^4^. We should collectively be encouraged to assess output using a much broader approach, for which an increasing number of tools is becoming available, including online resources such as Google Analytics ( https://analytics.google.com/) or Google Scholar ( https://scholar.google.com/), Altmetric ( https://www.altmetric.com/), author-specific metrics such as h-index, and – most importantly - the application of common sense to viewing and interpreting all metrics in the right context ^12– 14^.

### Authorship

In clinical medicine, powering studies sufficiently to answer relevant questions often requires the recruitment and analysis of huge multicentre cohorts; similarly in scientific studies there are increasing examples of ‘team science’. Acknowledging each individual contributor can be challenging, but the crucial importance of this is highlighted in a report by the Academy of Medical Sciences ^15^. A reciprocal problem can also arise, whereby individuals are listed as authors despite not having made substantial contributions to a project. Many journals now try to address this by asking for specific details of the contributions made by each individual to the final piece of work.

### Open access

Open access publication offers a system that should be inherently fair in promoting free access to published resources. However, a major challenge to equity here is an economic one ^16^. In a traditional, non open access model, the fees required for access to a journal or individual manuscript are frequently prohibitive for individuals; access therefore depends on institutional subscriptions. In the open access model, in order to make the work freely accessible to their readers, the publisher passes the costs on to their authors. Both systems discriminate strongly against those in less affluent settings.

Unsurprisingly, open access publication can influence article metrics, as those articles that are freely available may be more frequently cited ^17^. So authors from wealthy institutions can potentially feed their own personal metrics by publishing their work in open access fora. In reality, the situation is more complicated, as the open access citation advantage is inconsistent between studies ^18^, many publishing houses waive fees for authors from under-resourced settings, and there are now increasing options for free data sharing.

### Boundaries

An academic manuscript usually has to be assembled into a standardised package that meets strict formatting requirements; this may help individual publishers or journals with quality control, and with preservation of a unique identity through a ‘house style’. However, academics often see the formatting process as a complicated and time-consuming array of obligations, demanded of them before the work has even been accepted for publication, and without any appreciable benefit to quality ^3^. Among publishers, a more diverse body of opinion is reflected between those who are in favour of relaxing (or unifying) formatting requirements and those who do not feel any change is required ^4^. Online publication should progressively be providing an escape route from these constraints – albeit not one that has been consistently deployed or accepted.

Another, broader, boundary is also in operation – that which governs so strictly the fundamental nature of a piece of work, that which inhibits (or even prohibits) publication of a work-in-progress, or an unproved hypothesis, or results that are negative, unexplained or in conflict with previous data. Only 9% of academics agreed with the statement ‘the process of publication is flexible, supports innovation, and allows me to creative’, and none strongly agreed ^3^.

This should be of significant concern as there is increasing recognition of the risks and costs associated with the suppression of negative results ^19, 20^. Furthermore, when innovation underpins so much true scientific progress, why are such tight restraints imposed on the nature, style, content and substance of academic output? We should move towards a system that welcomes diversity: there is much for us all to gain from encouraging dissemination of a wider body of work. This might include new concepts, methods and strategies, diverse commentary and critique, approaches that have been tried and failed, negative results, unfinished projects, protocols and registries for clinical trials, and live datasets that evolve over time.

The traditional publication of an academic ‘paper’ makes it impossible to add incremental advances or updates, and the only way to correct inconsistencies that emerge post-publication is to submit and publish a formal erratum. This is a substantial missed opportunity for quality improvement. The version control option offered by newer publishing platforms allows authors to update their work to maintain it in its optimum form, while still preserving records of the original submission. This is the approach I have been ultimately been able to pursue for my own data, via the Wellcome Open Research platform ( https://wellcomeopenresearch.org/) ^21^, making it possible for this data resource to be updated and refined over time.

## Future challenges

### The resource gap

A publishing process perceived as equitable by one individual or institution may not operate in the best interests of another; this is particularly epitomized by the resource gap between different settings. Real fairness means reallocation of resources, waivers for institutions unable to pay access or publishing fees, better sharing of skill sets, balanced review, and capacity building in resource-limited settings ^22^.

### Maintaining quality

Diminishing or diluting quality is a potential concern as we enter an era in which a greater number of authors release a more diverse pool of work without pre-publication review. However, it is likely that market forces will tend to operate to maintain quality, and that the overall benefits of increasing data availability substantially outweigh any detriment to quality ^23^. The ‘author pays’ model can encourage publishers to accept submissions that fail to achieve a quality benchmark, on the grounds of the financial revenue accrued by their journal on acceptance of a submission ^24^; this highlights the importance of vigilance in ensuring that appropriate and consistent peer review is undertaken.

### Changing behaviour

New approaches to data sharing can be met with suspicion or opposition ^5^. Many authors are (either overtly or subliminally) wedded to the idea of a journal based impact factor and to blind peer review. Some authors also express anxiety arising from the potential conflict between wanting to share their output yet needing to retain ownership of the work. Substantial power is still held by a small subset of traditional journals and editorial boards who are keen to maintain the *status quo*, exerting an influence that – at times – can be ‘toxic’ ^25^.

### Predatory journals

Vigilance is required for so-called ‘predatory’ journals ^26^ that often send unsolicited emails trying to entice authors with offers including rapid and open access publication, but that quote flawed or misleading metrics, have an unskilled editorial board, fail to provide suitable peer review, and/or publish the work only on receipt of a substantial fee ^24, 26, 27^. An apparent explosion in the numbers of such enterprises is a threat to *bona fide* publishers, exploits authors and funders, and diminishes the quality of published science. All publishing stakeholders should seek to avoid interaction with these unscrupulous publishers and remove them from the academic record ^24^.

### Economic cost of publishing

I have not set out to include detailed discussion of economic cost, but it is clear that financial investment is crucial to support innovative approaches to publishing. Academia has to be willing to accept and underwrite these costs, and the publishing industry to develop a system that is lean and competitive, and that offers value for money.

## Caveats to this work

The discussions represented here took place over a short time frame and are based on opinions collected from a small section of academia ^3^ and from an even smaller slice of the publishing fraternity ^4^. Taking the opportunity to share feedback from academic clinicians does not mean that I represent all academic clinicians, or that the views of other sectors of academia are congruent.

## Conclusions

We are in an era in which the process of disseminating scientific work is evolving fast and undoubtedly for the better. Working towards improvements, and finding solutions to problems, is a dynamic process that evolves over time as a consequence of input and innovation from a wide number of sources, both within academia and publishing.

As well as promoting the FAIR principles, we should aspire to a process that is genuinely *fair* and underpinned by open and collaborative discussion at all stages of the process. There are wide-reaching benefits of fairness in publishing, which are pertinent to all the key stakeholders (summarized in Table 1, below). Our end goals should be the timely output of robust and high quality science, that is appropriately scrutinized by equitable peer review, and that can be shared, reproduced and collectively applied for the advancement of understanding.

**Table 1.** How and why we should strive for fairness in academic publication on behalf of a variety of stakeholders.

Stakeholder	Aspirations and rationale for fairness in academic publishing
**Authors**	• Reducing delays in publication to advance progress of individuals, teams, and the global scientific community; • Relaxing stringent formatting requirements to make the process less restrictive, onerous and time-consuming; • Optimizing peer review (e.g. open access reviewers, ensuring reviewers have appropriate expertise, minimising delay); • Providing open access portals, allowing work to be disseminated, used and cited widely; • Embracing diversity of output, including encouraging publication of negative results, experimental protocols, and unfinished datasets; • Reducing barriers to researchers in resource-limited settings; • Optimizing communication with publishers to make the process collaborative and efficient, and to drive ongoing improvements.
**Publishers**	• Representing publishers as an essential component of the process of data dissemination, and recognizing their role as a major driver for innovation; • Enhancing dialogue around cost; making data dissemination cost-effective while maintaining the viability of publishing businesses; • Developing a wider repertoire of output in order to meet the requirements of academia; • Optimizing communication with academia to make the process collaborative and efficient, and to drive ongoing improvements; • Identifying ‘predatory’ journals as harmful, and removing these from the academic record.
**Funders and academic** **institutions**	• Providing full and timely recognition of scientific output hosted or funded by specific agencies; • Developing opportunities for repositories of work produced by their researchers; • Providing feedback to stakeholders and investors; • Enhancing potential for collaboration; • Driving opportunities for innovation and translational output.
**Reviewers**	• Ensuring that reviewers have sufficient and appropriate expertise; • Providing formal credit and recognition of the contribution of reviewers.
**Patients**	• Acknowledging and rewarding the commitment and altruism of patients who enroll in clinical research; • Making results and conclusions of scientific research available to patients, their health- care teams, and those who allocate resources; • Avoiding harm through suppression of negative results.
**Public**	• Assuring accountability of public money; • Engaging and educating the public about science; • Adding to the resources available to educational institutions; • Making data available to other relevant agencies (e.g. the government, the media, economists, biotechnology).
