# Supplementary material for: Fairness in scientific publishing
Source: F1000Res. 2017 Jan 17;5:2816. Originally published 2016 Dec 5. [Version 2] doi: 10.12688/f1000research.10318.2 (PMC5247776; doi:10.12688/f1000research.10318.2)
Supplement: Peer review history

# Peer review: Referee response for version 2

**DOI:** 10.5256/f1000research.11479.r19407

**Published:** 2017-01-17

**Authors:** Dragan Pavlović

**Affiliations:** 1 Department of Anesthesia, Pain Manahement & Perioperative Medicine, Dalhousie University, Halifax, NS, Canada

I fully approve this corrected version of the manuscript.

 Indeed, not all important issues could ever be treated in a single article. I therefore want to point out here that stigmatizing the so called “predatory journals” is quite unfortunate part of the competition for the scientific journal markets and a result of the counter attack of the big journals that are losing markets. Before some fair rating of the scientific journals would be invented, the only reliable criterion must remain the scientific quality of each particular article itself and the arbitrary and a priori discrimination of some open access journals or "predatory journals" abandoned. If the authors would agree, I would suggest that they express their opinion on this issue in their article.

I have read this submission. I believe that I have an appropriate level of expertise to confirm that it is of an acceptable scientific standard.

# Peer review: Referee response for version 2

**DOI:** 10.5256/f1000research.11479.r19405

**Published:** 2017-01-17

**Authors:** Gustav Nilsonne

**Affiliations:** 1 Department of Clinical Neuroscience, Karolinska Institute, Stockholm, Sweden

In the last review, my main concern was that survey data should be published openly in a repository. This has been done. I note in passing that additional value could be added by publishing a data frame of individual participant responses, rather than response distributions for each question, as it would then be possible to independently analyse data for the purposes of validation or exploration. 

 The text has been improved in this revised version. The addition of Table 1 to summarise key concerns is particularly helpful. 

 I am happy to approve this submission.

I have read this submission. I believe that I have an appropriate level of expertise to confirm that it is of an acceptable scientific standard.

# Peer review: Referee response for version 1

**DOI:** 10.5256/f1000research.11114.r18988

**Published:** 2017-01-16

**Authors:** Oyewale Tomori

**Affiliations:** 1 Nigerian Academy of Science, Academy House 8A Ransome Kuti Road, University of Lagos, Akoka, Yaba Lagos, Nigeria

The title for this article is appropriate.

 The abstract adequately summarises the article. In addition, the article goes beyond the FAIR acronym (Findable, Accessible, Interoperable and Reusable), for authors and publishers and addresses FAIRNESS on equity and justice for patients, clinicians, academics, publishers, funders and academic institutions. This is an opinion and not an "opinionated" paper. It is backed by a succinct and balanced analysis of responses to distributed questionnaire and discussions with stakeholders in publishing **,** authors and editors.

 The conclusions are balanced and justified on the basis of the results.

 I have also made some additional comments to the manuscript. To see this, please click here.

I have read this submission. I believe that I have an appropriate level of expertise to confirm that it is of an acceptable scientific standard.

# Peer review: Referee response for version 1

**DOI:** 10.5256/f1000research.11114.r19008

**Published:** 2017-01-06

**Authors:** Dragan Pavlović

**Affiliations:** 1 Department of Anesthesia, Pain Manahement & Perioperative Medicine, Dalhousie University, Halifax, NS, Canada

General description.

 This is a well written “opinion” article where the author examined the parallel view that “we should take a collective stance on making the dissemination of scientific data *fair* in the conventional sense, by being mindful of equity and justice for patients, clinicians, academics, publishers, funders and academic institutions.” The views are based on oral and written dialogue (including 2 online questionnaires, 102 academics and 37 representatives of the publishing industry,) with clinicians, academics and the publishing industry. Parts of the work were presented earlier at 2 meetings. It is concluded that further progress is needed to improve collaboration and dialogue between these groups, to reduce misinterpretation of metrics, to reduce inequity that arises as a consequence of geographic setting, to improve economic sustainability, and to broaden the spectrum, scope, and diversity of scientific publication.

  

- Major comments.  

This is in some way a relatively “short” text, mostly presented as a letter or a superficial comment, yet as such it appears to be quite long. There is no precise analysis of the announced results of the 2 online questionnaires, with 102 academics and 37 representatives of the publishing industry. The text remains to be just, as indeed announced, an opinion.

 It looks to me that the text could be much shorter and much more focused on the acute problems, like expertise of the peer reviewers, negligence of the editors and the editorial boards of the journals, co-authorship and commercialization of the open access journals (‘predatory’ journals). Probably also the last paragraphs (Future challenges and Conclusions) could be substantially shortened. Or, if the questionnaires were appropriate, it would be possible to develop much more relevant and informed study. It is hard to see what how the study would look like since the questionnaires are not available.

 The explosion of the number of the journals worldwide in the last decade or so was not discussed and there is no mention of the problem with the printed journals that are facing their slow disappearance.

 The discussion does not reach deep enough to provide more concrete solutions to the problems that are presented in the paper.

  

- Minor comments 

**Peer review**

 Insisting on the expertise of the reviewers is justified, although the existing methods - some are mentioned in the text, do not guarantee it. It should be mentioned that the journals should have some more secure methods to choose the relevant experts for the peer review. May be the reviewers should supply some evidence what kind of the expertise they have in the relation to the paper that they give an opinion and the journals should be obliged to respect it.

**Metrics**

 Problem of co-authorship and possible unjustified benefits for the co-authors was not mentioned.

**Open access**

 Problem of commercialization (of the ‘predatory’ journals) could be more elaborated.

**Formatting requirements**

 Probably some negative comments are not fully justified. I personally find impossible to review an article that, even if well written, is badly formatted. Badly presented text, even if it is of high quality, inevitably loses its impact. Please revise if you agree that your judgment was not carefully measured.

I have read this submission. I believe that I have an appropriate level of expertise to confirm that it is of an acceptable scientific standard, however I have significant reservations, as outlined above.

# Peer review: Referee response for version 1

**DOI:** 10.5256/f1000research.11114.r18242

**Published:** 2016-12-07

**Authors:** Gustav Nilsonne

**Affiliations:** 1 Department of Clinical Neuroscience, Karolinska Institute, Stockholm, Sweden

This paper is an opinion article, which discusses several areas where unresolved questions exist in the transition to more open scientific publication practices. The discussion is underpinned by survey data, although a full description of the survey and its results are not within the scope of the paper.

 Areas covered in this paper are publication delays in scientific publishing, peer review, communication between scientists and publishers, metrics such as the impact factor, models for open access publication, journals' formatting requirement, and boundaries imposed by traditional publishing on paper, which need not persist in a time with online publishing, but still do.

 The paper provides a timely discussion, based on survey data, and on relevant and valid arguments. The abstract promises to explore the notion of fairness from the points of view of many different stakeholders. This point of departure lacks clear justification. In particular, it is not obvious why changes in scientific publishing should need to be perceived as fair by publishers. Also, not all of the stakeholder perspectives are explicitly addressed in the main text.

 The survey data are available in two linked slide presentations. I recommend that the data be made available as a data frame in a non-proprietary file format. This will facilitate re-use and further exploration of the data set. Best practice is to use a repository that provides access to data in a format that is time-stamped, immutable, and permanent, and with a persistent identifier and an open licence. Documentation including metadata that describes how the survey was performed can be provided with the data or in this paper.

 In the current movement towards more open publication practices, it is important to find out how scientists and other stakeholders perceive barriers and possibilities. This paper makes a valuable contribution in gathering scientists' views and arguments surrounding publication practices. I am happy to approve it with a reservation about the format of openly published survey data.

I have read this submission. I believe that I have an appropriate level of expertise to confirm that it is of an acceptable scientific standard, however I have significant reservations, as outlined above.

# Response

**Published:** 2016-12-22

**Authors:** Philippa Matthews

**Affiliations:** Nuffield Department of Medicine, Peter Medawar Building for Pathogen Research, University of Oxford, UK

Thank you Dr Nilsonne for the positive feedback and helpful critique.

 I have uploaded the metadata to Oxford University Research Archive; this record can be accessed using the following link: https://doi.org/10.5287/bodleian:J5aekGAMy.

 I will address the other suggestions in more detail in a revised version of the article.
